# Post-translational modifications of silk proteins

**DOI:** 10.1039/d6cb00012f

**Published:** 2026-03-05

**Authors:** Kota Nomura, Keiji Numata

**Affiliations:** a Department of Material Chemistry, Graduate School of Engineering, Kyoto University Kyoto 615-8510 Japan keiji.numata@riken.jp; b Biomacromolecules Research Team, RIKEN Center for Sustainable Resource Science Saitama 351-0198 Japan

## Abstract

Post-translational modifications (PTMs) endow silk proteins with chemical diversity that governs their higher-order assembly, hydration, and covalent connectivity. This review highlights the principal PTMs that define silk protein function, including hydroxylation, glycosylation, phosphorylation, and covalent crosslinking. We also describe their contributions to protein structural stability and mechanical properties. Recent advances in proteomics have begun to reveal low-abundance PTMs, whereas synthetic biology and bioorthogonal chemistry enable the programmed installation of modifications to tune physicochemical properties. Understanding and harnessing these chemistries provides a foundation for the predictive design of next-generation protein-based materials at the interface of chemical biology and materials science.

## Abstract 2

Post-translational modifications (PTMs) endow silk proteins with chemical diversity that governs their higher-order assembly, hydration, and covalent connectivity.

## Graphical abstract

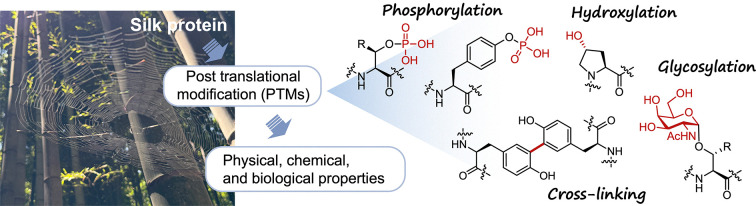

## 1. Introduction

Silk proteins are fibrous structural biomacromolecules produced by diverse organisms, including spiders, silkworms, and caddisflies, and are well known for forming fibers with exceptional mechanical performance, such as high tensile strength, toughness, and environmental resilience.^1–3^ These properties arise from the hierarchical organization of silk proteins, spanning amino acid sequence design, self-assembly, and higher-order structural transitions during fiber formation.

Importantly, the performance of silk fibers is governed not only by their primary amino acid sequences but also by the chemical diversity imparted by post-translational modifications (PTMs),^4^ such as phosphorylation,^5,6^ proline hydroxylation,^7^ and dityrosine cross-links.^8^ By modulating intermolecular interactions, hydration states, higher-order folding, and fibril assembly, these PTMs play critical roles in directing silk protein assembly and tuning the final mechanical and functional properties of the resulting materials.

In this review, we summarize PTMs identified in silk proteins and discuss how these modifications contribute to silk-material properties. Integrating biochemical, structural, and materials-science perspectives, we highlight silk PTMs as a distinctive class of modifications that bridge molecular chemistry and macroscopic material function.

## 2. Classification of PTMs in silk proteins

### 2.1. Hydroxylation

**Fig. 1:**
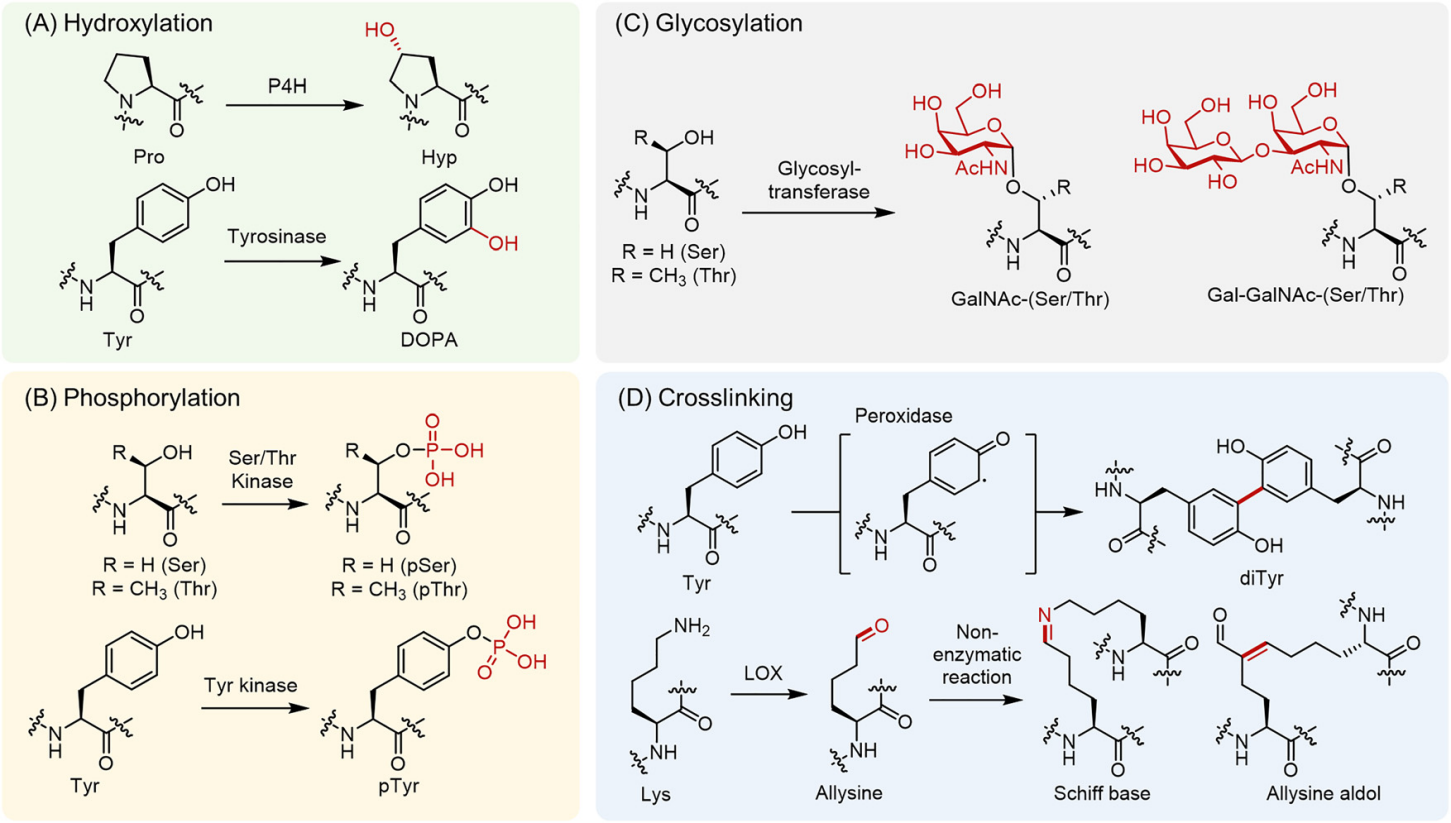
Structures of representative PTMs of silk proteins. Panel A shows hydroxylation reactions, including the conversion of Pro to Hyp by P4H and Tyr to DOPA by tyrosinase.^9–15^ Panel B depicts phosphorylation, in which the Ser/Thr hydroxyl group or the phenolic group of Tyr are modified by protein kinases.^16,17^ Panel C illustrates *O*-glycosylation, including the addition of simple sugars such as GalNAc, Gal-GalNAc to Ser/Thr.^18,19^ Panel D presents cross-linking reactions, including peroxidase-mediated diTyr formation and the LOX-mediated oxidation of Lys to allysine.^20–23^

Enzymatic hydroxylation is a hallmark PTM of silk proteins (Fig. 1A).^9^ Prolyl 4-hydroxylase (P4H) converts certain Pro residues to 4-hydroxyproline (Hyp).^10^ These enzymes reside within the lumen of the rough endoplasmic reticulum (ER) and act on nascent polypeptides. The Hyp residues introduced by ER-localized P4H greatly stabilize fibrillar architectures *via* strengthened hydrogen bonding interactions and increased hydration.^11^ In some structural or adhesive contexts, tyrosine (Tyr) residues are hydroxylated to 3,4-dihydroxyphenylalanine (DOPA).^12,13^ This reaction is catalyzed by phenoloxidase enzymes such as tyrosinase or insect tyrosine hydroxylase in the secretory pathway or extracellularly.^13,14^ The resulting DOPA provides a catechol functional group that enables metal coordination and redox-driven curing, as in marine mussel adhesives.^15^

### 2.2. Phosphorylation

Serine/threonine (Ser/Thr) protein kinases introduce phosphate groups to yield phosphoserine (pSer) or phosphothreonine (pThr).^16^ Similarly, Tyr kinases introduce phosphate groups to produce phosphotyrosine (pTyr) (Fig. 1B). In proteins, phosphorylation typically occurs after translation in the secretory pathway, for example, by a Golgi-resident kinase.^17^ The fibroin heavy chain of the silkworm *Bombyx mori* contains multiple pSer residues in its repeat regions,^5^ and in spider silk,^6^ phosphorylation of the terminal domain is observed.^8^ In silk fibroin, phosphorylation is thought to modulate the storage solubility of the protein dope (the highly concentrated aqueous silk-protein solution stored in the silk gland prior to spinning) and to bias β-sheet formation during spinning. Changes in phosphorylation status act as a reversible switch, maintaining solubility in the gland prior to spinning and promoting assembly during spinning.

### 2.3. Glycosylation

Glycosylation generally occurs in two main forms, with *O*-linked glycosylation occurring on Ser/Thr residues and *N*-linked glycosylation occurring on Asn residues. *N*-Linked glycosylation is less frequently highlighted in the context of silk proteins, as *O*-linked modifications are more prominent^18^ (Fig. 1C). For example, *O*-glycosylation, such as in the saccharides α/β-d-*N*-acetyl galactosamine (GalNAc) and glucosamine (GlcNAc), occurs at Ser and Thr residues on certain adhesive proteins, such as aggregate silk spidroin.^19^ Glycosylation contributes to the hydration and adhesion properties of the substrate.

### 2.4. Enzymatic crosslinking

Lysyl oxidase (LOX) is a Cu-dependent amine oxidase secreted into the extracellular space as a proenzyme that is activated outside the cell (Fig. 1D).^20–22^ It acts post-translationally in the extracellular matrix (ECM) by oxidatively deaminating Lys residues to yield reactive aldehydes (*e.g.*, allysine), which can form covalent cross-links through spontaneous condensation reactions, including Schiff base formation with Lys residues and aldol condensation with other allysine residues.^23^

### 2.5. Oxidative modifications

Oxidative coupling of Tyr side chains yields dityrosine (diTyr) and trityrosine (triTyr) cross-links (Fig. 1D).^24,25^ Such cross-links can form nonenzymatically upon interaction with reactive oxygen species, but in biological systems, they are often formed enzymatically by oxidases (*e.g.*, peroxidases or laccase-type phenoloxidases) secreted into the ECM.^26^ In insect cuticles, resilin, and fibroin, peroxidase oxidizes Tyr residues to form Tyr radicals that couple to form diTyr cross-links.^27^ These oxidative Tyr linkages are integral to the unique elastic recovery of resilin, effectively creating an amorphous rubber-like network.^28,29^

### 2.6. Other nonenzymatic modifications

A variety of spontaneous chemical degradation products accumulate over time and with environmental exposure. Examples include deamidated asparagine and glutamine (Asn/Gln) residues,^30^ oxidized methionine (Met) to sulfoxide,^31^ and disulfide bond reshuffling or cleavage of cysteine (Cys) residues.^32^ These products are formed in the presence of physicochemical stimuli (pH, concentration, reactive oxygen species, and UV irradiation). These changes are typically detrimental to material performance. For instance, deamidation alters the charge and conformation of the protein, leading to protein brittleness, and Met oxidation or unwanted disulfide changes in Cys can reduce protein elasticity or stability.^33^

## 3. PTMs in silk proteins and their function

### 3.1. PTMs in spider silks

#### 3.1.1. Major spider silks (major ampullate)

**Fig. 2:**
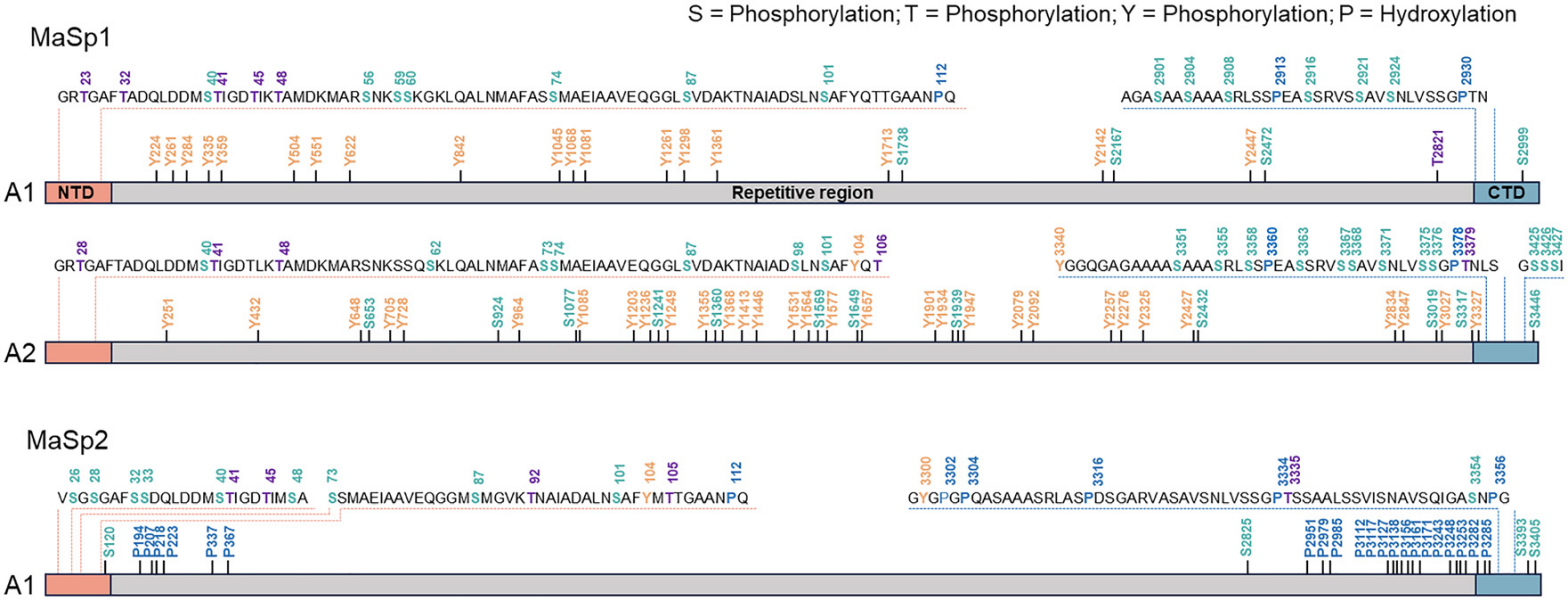
Comprehensive map of the PTMs of MaSp1 and MaSp2 of *Trichonephila clavata*. The numbered residues correspond to phosphorylated Ser (S, light green), Thr (T, purple), or Tyr (Y, orange) residues and hydroxylated Pro residues (P, blue). NTD, N-terminal domain; CTD, C-terminal domain.^6^ Adapted from ref. 4 under the Creative Commons CC BY 4.0 license.

**Fig. 3:**
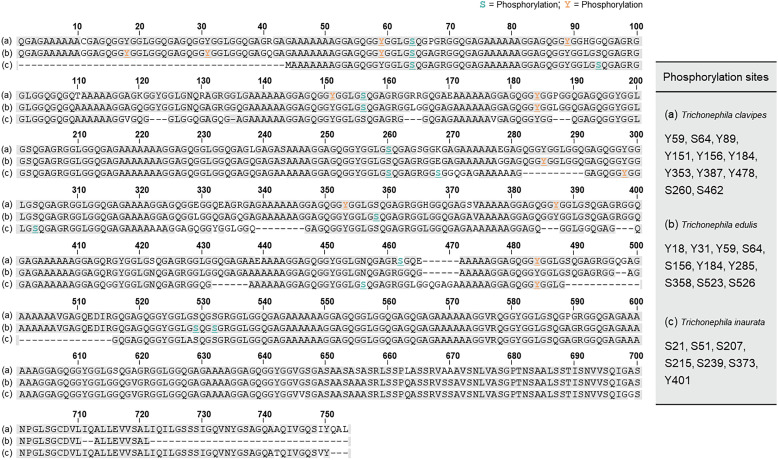
Multiple alignment of the primary sequences of MaSp1 from (a) *Trichonephila clavipes*, (b) *Trichonephila edulis*, and (c) *Trichonephila inaurata*. The numbered residues correspond to phosphorylated Ser residues (S, light green) and phosphorylated Tyr residues (Y, orange). PTMs of MaSp1 revealed by MASCOT.^8^ Adapted with permission from ref. 8 Copyright 2016, Elsevier.

Recent mass spectrometry (MS) studies have revealed diverse PTMs in representative spider silks, including dragline silk, extensible flagelliform silk used for prey capture, and adhesive glue droplets from the aggregate gland.^6,34–37^ In the large structural proteins of dragline silk, phosphorylation of the major ampullate spidroin (MaSp) is particularly prominent.^8,35,38^ For example, in the Jorō spider (*Trichonephila clavata*), numerous phosphorylated Ser, Thr and Tyr residues have been identified across MaSp1-3, including sites concentrated in the *N*-terminal domain (NTD) at the dimer interface (Fig. 2).^6^ Among *Trichonephila* species, the number of MaSp1 phosphorylation sites varies from 7 residues (*T. inaurata*, formerly *Nephila madagascariensis*) to 10 (*T. edulis*, formerly *N. edulis*) and 11 residues (*T. clavipes*, formerly *N. clavipes*) (Fig. 3).^8^ In addition, diTyr cross-links are consistently detected, and trace amounts of DOPA have been reported in some studies.^8^ Within the MaSp repetitive regions, numerous pSer sites have been identified, several of which are positioned upstream of hydrophobic poly-Ala crystalline runs, where phosphorylation has been proposed to act as a flexible β-sheet terminator and may disrupt β-strand propagation (*e.g.*, GpSAAAAAAG).^6^ Phosphorylation of the NTD of MaSp1/2 is concentrated at the dimer interface and can shift the pH midpoint (pKa) of dimer formation in phosphomimetic Asp variants, suggesting that phosphorylation may influence NTD assembly.^39^ Consistent with these observations, pSer/pThr near poly-Ala residues or within repeat regions has been associated with reduced crystallinity in MaSp-based systems and has been proposed to contribute to solubility maintenance and reduced premature aggregation during spinning. Additionally, Hyp has been detected in dragline silk. In draglines of *Argiope keyserlingi* and *T. clavata*, Hyp is present within Pro-rich repeats (*e.g.*, GPGXX). Solid-state NMR of MaSp2 of *Argiope aurantia* revealed that GPGXX motifs adopt elastin-like β-turns,^7^ whereas in *A. keyserlingi*, DNP-enhanced solid-state NMR revealed the presence of Hyp and suggests that the additional hydroxy groups can participate in hydrogen bonding within β-spiral segments, potentially stabilizing local turns.^40^ In contrast, *T. clavata* spectroscopy indicated that Hyp can disrupt β-turns and favor more disordered conformations.^6^ By shifting these Pro-rich segments away from turn-mediated ordering, Hyp might help limit overcrystallization and support toughness. In summary, PTMs in dragline silk finely tune molecular self-assembly and mechanical properties. However, quantitative analyses of phosphorylation stoichiometry and site occupancy in native spider silks remain limited, and future studies integrating quantitative proteomics with structural and mechanical analyses will be essential to elucidate their functional significance.

#### 3.1.2. Flagelliform (prey-capture) silk

**Fig. 4:**
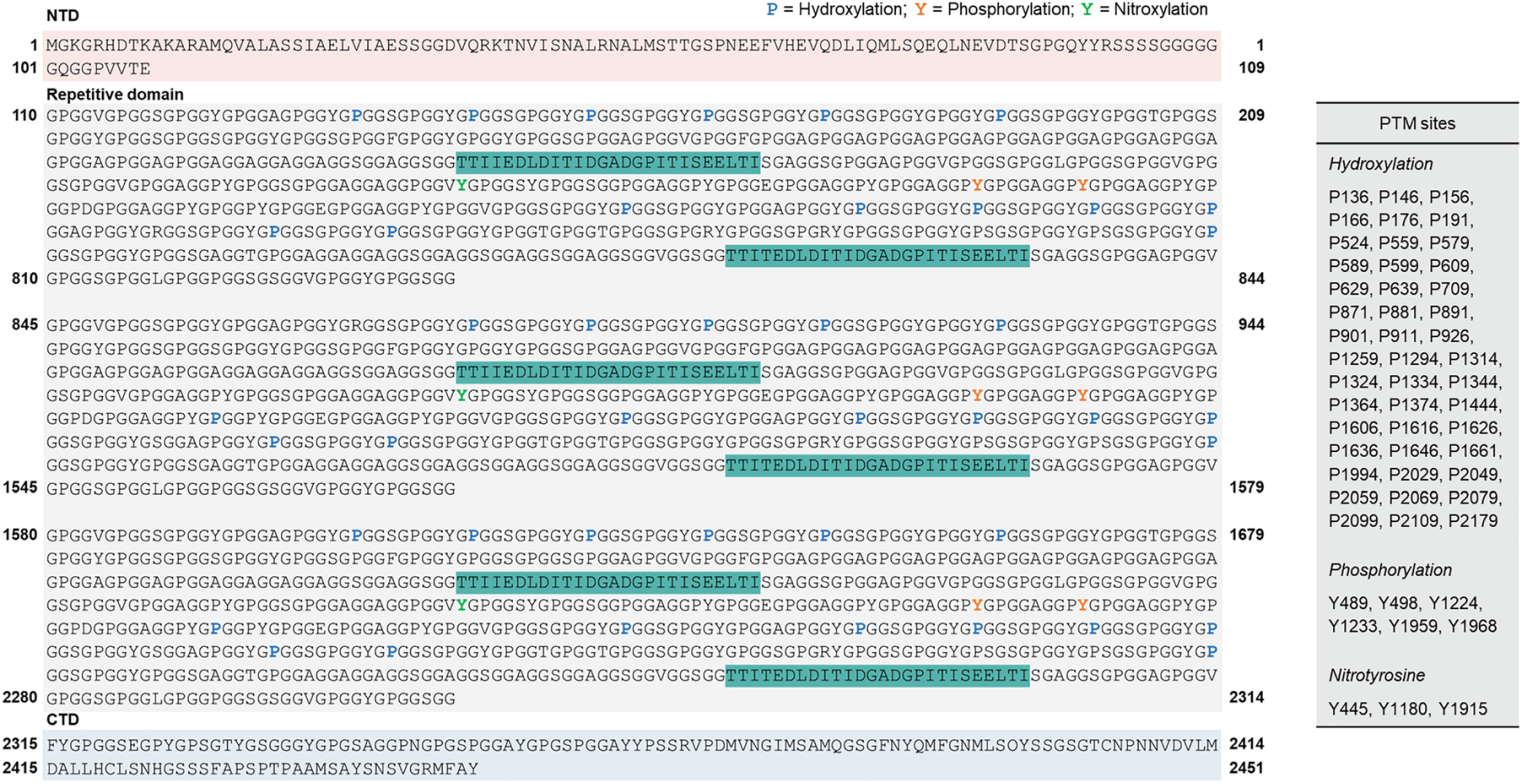
Representative sequences of flagelliform silk from *Trichonephila clavipes.* The numbered residues correspond to phosphorylated Tyr residues (Y, orange) and hydroxylated Pro residues (P, blue); the spacer domain is indicated in green. The NTD domain is indicated in light red. The repetitive domain is indicated in gray. The CTD domain is indicated in light blue. PTMs of flagelliform silk revealed by MASCOT.^39–41^ Adapted from ref. 41 under the Creative Commons CC BY 4.0 license.

Flagelliform silk, characterized by Gly- and Pro-rich repeats (GPGGX) and extreme extensibility, also exhibits distinctive PTMs. It has been reported that more than 45 Pro residues within these repeats are converted to Hyp (Fig. 4).^41^ Such hydroxylation was previously anticipated from sequence analyses^42^ and structural models^43^ and provides additional hydrogen-bonding capacity within the repetitive motifs. Hyp incorporation is widely believed to facilitate the formation of spring-like β-spirals, as the altered hydrogen-bonding network in GPGGX repeats could contribute to elastic recovery and supercontraction.^43,44^ Indeed, flagelliform exhibits pronounced contraction with changes in humidity,^45^ and the abundance of Hyp is thought to modulate hydration and stability.^46^ Phosphorylation in flagelliform has also been confirmed. MS and immunoblotting revealed multiple phosphorylation events, mainly on Ser/Thr, with evidence for Tyr phosphorylation at several positions, although the precise number and functional importance of these events remain to be clarified.^41,47^ Nitrotyrosine has likewise been detected. As a product of reactive nitrogen species, nitrotyrosine might affect crosslinking and interprotein interactions within the fiber.^41^ Flagelliform is regarded as unusual in that it does not undergo structural conversion during spinning, making PTMs especially important for stabilizing pre-existing structures and tuning mechanical properties. These PTMs also contribute to the remarkable energy absorption and extensibility of the capture spiral, where hydration-dependent conformational changes are directly coupled to macroscopic mechanics.

#### 3.1.3. Aggregate gland (glue) silk

The sticky adhesive droplets secreted onto prey-capture threads derived from the aggregate glands and are composed of specialized silk proteins (AgSps), whose major components are glycoproteins.^48–50^ The principal glue protein is AgSp1 (formerly reported as ASG2), a spidroin-type glycoprotein expressed specifically in the aggregate glands.^48,50–52^ Earlier work also identified ASG1, but subsequent genomic and transcriptomic analyses demonstrated that ASG1 belongs to a peritrophic matrix-like mucin family and is not a glue component.^48,50^ AgSp1 contains abundant Ser/Thr residues that are extensively *O*-glycosylated, predominantly with GalNAc moieties, conferring hygroscopicity and preventing premature curing. These glycans form a hydrogel-like matrix that retains water and remains intact even under saturated humidity.^48,53,54^ Phosphorylation has also been reported in glue proteins. Previous studies have indicated that AgSp glycoproteins contain abundant pSer/pThr residues, and these negatively charged phosphate groups are proposed to enhance water absorption and retention.^55–57^ Remarkably, spider glue becomes more adhesive as the humidity increases and retains its performance even at nearly 100% relative humidity, which is rare among natural adhesives.^50^ This is attributed to a hydrogel-like matrix formed by dense glycans and phosphorylated residues, which bind ambient water and optimize the glue's tackiness under damp conditions.^55^ Indeed, glycoprotein glue behaves as a viscoelastic solid that can take up moisture without losing cohesion, thereby enhancing adhesion in wet environments.^54^ It has even been proposed that pSer coordinates divalent cations (*e.g.*, Ca^2+^) to form transient cross-linking networks that can stabilize adhesion under humid conditions.^58^ Thus, aggregate gland-derived adhesive silk achieves its stickiness through *O*-glycosylation and phosphorylation.

#### 3.1.4. PTMs of minor gland silks

Beyond the major silks above, spiders possess several special-purpose silks secreted from smaller glands, such as tubuliform silk (TuSp; egg case silk), aciniform silk (AcSp; wrapping silk), and piriform silk (PiSp; attachment silk).^59–61^ Each differs in function and composition, and knowledge of their PTMs remains limited.

Tubuliform silk in the outer egg sac layer is characterized by a very high Ser content and low Gly content relative to those of other spider silks.^60^ This distinctive amino acid profile is thought to account for its propensity to form rigid fibers through extensive β-sheet crystallization. Given the high Ser content, it is plausible that the principal egg sac proteins harbor pSer. However, definitive evidence of PTMs is lacking. For example, proteomic studies on the egg sac silk of *Latrodectus hesperus* have identified TuSp1 as the major fibroin together with additional egg case proteins (ECPs), but evidence of phosphorylation or other modifications has not yet been reported.^62,63^ Even so, because the egg sac presents a robust barrier that protects eggs from UV radiation and microbes, it has been hypothesized that diTyr cross-links occur after egg sac formation to stabilize the fibers. Evidence for such oxidative crosslinking and pigmentation has been reported in silkworm egg sacs and cocoons.^64,65^

Piriform silk is a specialized protein that provides strong adhesion between silk threads and substrates. Rather than being a thread, it is secreted as a pad-like deposit and must harden and set extremely quickly.^61^ However, there is currently no direct evidence of PTMs in PiSp proteins.

The role of the aciniform silk in enveloping prey and lining the inner layer of the egg sacs suggests a balance between extensibility and moderate strength.^59^ Aciniform fibroins possess Gly/Ala-rich repetitive motifs akin to those in dragline silk. Some analyses have reported a slightly greater proportion of acidic residues in aciniform silk than in dragline. It was shown that an aciniform spidroin (AcSp1) is a key component of the small diameter fibers in egg sacs as well as in prey wrap silk, emphasizing that aciniform silk contributes to both prey capture and protection.^59,66^ Recent research on *Argiope argentata* has shown that the dope adopts an α-helical beads-on-a-string conformation in which Ser/Ala/Gly-rich linkers adopt β-sheet structures upon spinning^67^ and that AcSp2 yields recombinant fibers extending to approximately 160%, underscoring sequence-level control of AcSp mechanics.^68^

### 3.2. PTMs in silkworm (*Bombyx mori*) silk

#### 3.2.1. Silk fibroin main chain

**Fig. 5:**
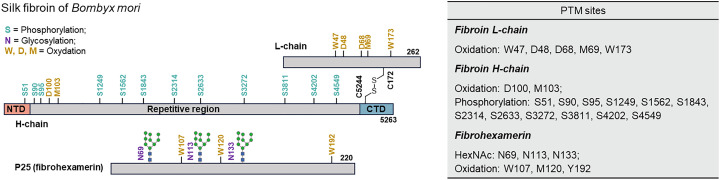
The modifications of silkworm silk fibroin from *Bombyx mori*. The numbered residues correspond to phosphorylated Ser residues (S, light green), N-glycosylated Asn residues (N, purple) and oxidized Trp, Asp, and Met residues (W, N, M, yellow). The NTD domain is indicated in light red. The repetitive domain is indicated in gray. The CTD domain is indicated in light blue. PTMs of silk protein revealed by MASCOT.^67,68,73^

The silk fibroin of the domesticated silkworm, *Bombyx mori*, is secreted as a complex of three components, namely, a heavy chain (H chain), a light chain (L chain), and fibrohexamerin (P25) (Fig. 5).^69,70^ The H-chain (molecular mass of approximately 350 kDa) forms a fiber-forming core dominated by Gly-Xaa repetitive motifs (with Xaa mostly Ala, Ser, and Tyr).^71^ Recent proteomics studies have revealed multiple phosphorylation sites on the fibroin H-chain.^5,72^ Such phosphorylation has been proposed to contribute to stabilization of the liquid crystalline fibroin gel within the silk gland and to influence the phase transition during spinning (the change from the structure of Silk I to Silk II).^5,73^ In other words, phosphorylation might suppress excessive β-sheet association and aggregation, allowing the fibroin to remain soluble even at high protein concentrations.^5^ In addition, other PTMs have been reported on the fibroin H-chain. Lubec and colleagues detected oxidative modifications of Lys (allysine) and Tyr cross-links in peptides derived from cocoon fibroin.^5^ These findings suggest that because the H-chain lacks Cys and Trp residues, such oxidative PTMs would act as alternative crosslinking routes that harden the fibers.^74^

#### 3.2.2. PTMs of the fibroin light chain, P25, and sericin

The fibroin L-chain (molecular mass of 25 kDa) forms a disulfide bond with the H-chain to construct the basic unit (molecular mass of 2.3 MDa) composed of six H-chains, six L-chains, and one P25 unit (Fig. 5).^75^ The L-chain itself is rich in Gly and Cys residues, bridging and stabilizing the C-terminal region of the H-chain, but its PTMs are limited.^70^ In its sequence, the L-chain contains an N-linked glycosylation motif (NXS/T); however, the L-chain is thought to function in a non-glycosylated form.^75^ More importantly, it plays a role in protecting the glycans of P25. P25 (approximately 30 kDa) stabilizes the fibroin complex and is a glycoprotein bearing multiple *N*-linked glycans, with three consensus sites (Asn69, Asn113, and Asn133). The glycans of P25 are of the high-mannose type and, together with binding to its hydrophobic core, fix P25 at the center of the H-chain and L-chain complex. Indeed, in the naked pupa mutant lacking the L-chain due to a defect in fibroin synthesis, a complex with a H-chain : P25 ratio of 6 : 1 still exists, but because the P25 glycans are exposed, most of it is degraded by quality control machinery. In the normal system where the L-chain is present, the glycans of P25 are masked by the L-chains, which both protects them from proteases and maintains the higher-order structure of the complex. Thus, *N*-glycosylation of P25 is essential for the formation of the fibroin unit, and interactions between its glycans and the L-chains likely contribute to the intracellular assembly and secretion of the large complex.^75^ Another interesting point about P25 is that multiple isoforms exist. These differences have been reported to arise not only from subtle variations in glycan structure but also from reversible phosphorylation events.

Sericins are another important group of proteins that make up the cocoon, functioning as both an adhesive and a protective coating for the silk filament.^76^ Sericins consist of multiple subunits of different sizes (Ser1, Ser2, Ser3, *etc.*), all of which are characterized by high contents of hydroxy amino acids and repetitive sequences. Sericins are secreted and deposited around fibroin in the middle section of the silk gland and harbor typical *O*-linked glycans (α-Gal and α-GalNAc).^76^ These glycans enhance the adhesive properties of the sericin, strengthening adhesion between the cocoon and surrounding twigs after spinning. A notable PTM of sericins is the formation of cross-links. In the domesticated silkworm, phenoloxidases are released immediately after cocoon formation; thus, domesticated sericin remains uncrosslinked within the cocoon and can be readily extracted with warm water. In contrast, in wild silkmoths, sericins sometimes undergo partial oxidative polymerization during cocooning or cocoon sheet formation, producing an insoluble, resin-like matrix. For example, in *saturniid* cocoons, laccase-like enzymes are secreted at the final larval instar, oxidizing the Tyr residues of sericin and exogenous tannins to form quinone crosslinks, thereby causing the cocoon to darken and become insoluble.

### 3.3. PTMs in caddisfly silk

#### 3.3.1. PTMs that support underwater adhesion

**Fig. 6:**
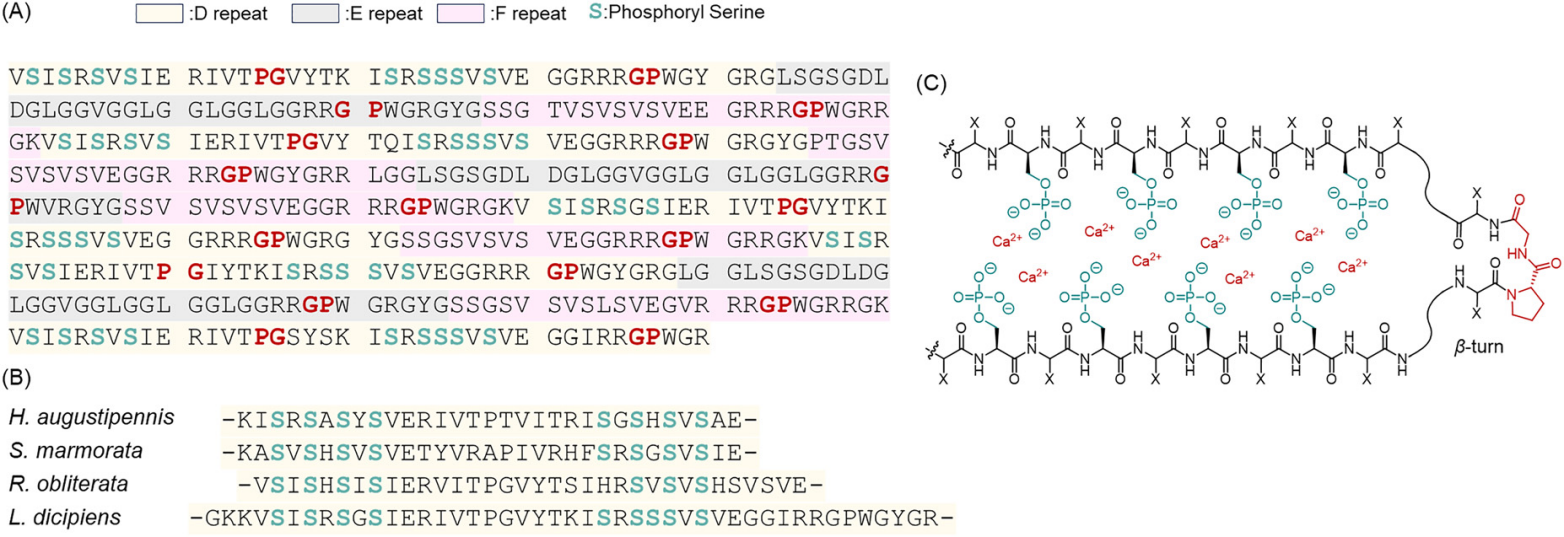
(A) Partial amino acid sequences of heavy-chain fibroin protein from the caddisfly silk. Phosphorylated (SX)_4_ motifs found in the D-repeat (shown in light yellow) are proposed to exist in calcium-phosphoserine sheets *via* pSer (light green). In support of the sheet model, proline residues often found in β-turn secondary structures appear shortly after every (SX)_4_ region. (B) Sequence correlation of D-repeat motifs across four distinct species of caddisfly (*Hydropsyche augustipennis*, *Stenopsyche marmorata*, *Rhyacophila obliterata*, and *Limnephilus decipiens*).^78^ Adapted with permission from ref. 78 Copyright 2013, American Chemical Society. (C) Schematic illustration of calcium–phosphate interactions within phosphorylated β-sheets.^79,80^ Adapted with permission from ref. 79 Copyright 2010, American Chemical Society.

Caddisfly larvae (order *Trichoptera*) include case-builders that glue stones and twigs together underwater to make protective tubular cases, as well as species that spin underwater capture nets.^77^ Caddisfly silk is phylogenetically similar to silkworm and moth silks; however, it is characterized by a distinct molecular strategy adapted to the aquatic environment.^78^ In contrast to spider and silkworm silks, phosphorylation in caddisfly silk occurs at unusually high stoichiometry, and constitutes a structurally significant component of the fiber architecture rather than a low-level modification. The fibroin heavy chain (H-chain), the principal component of caddisfly silk (molecular mass of 250–500 kDa), contains relatively few Ala and Gly residues but is rich in Ser and Thr residues.^79^ Instead of poly-Ala motifs, the caddisfly H-chain features a characteristic repetitive D-repeat consisting of (Ser-Xaa)_4_, where Xaa is primarily Val or Thr residues.^80,81^ Notably, analyses revealed that approximately 60% of the Ser residues in the H-chain are phosphorylated (Fig. 6A). Phosphorylation of these (SX)_4_ motifs within the D-repeat is a conserved feature reported across diverse species, including *Hydropsyche augustipennis*, *Stenopsyche marmorata*, *Rhyacophila obliterata*, and *Limnephilus decipiens* (Fig. 6B).^79,80^ According to Stewart and colleagues, caddisfly silk threads contain pSer, amounting to approximately 10 mol% of the total amino acid residues.^82^ Experimental evidence shows that abundant pSer plays a structural role by coordinating with metal ions (*e.g.*, Ca^2+^ or Fe^2+^) to stabilize the silk. X-ray diffraction (XRD) and solid-state NMR show that these pSer residues form complexes with multivalent cations to construct β-sheet nanocrystals stabilized by metal coordination.^80^ When metal ions are extracted from the fibers with ethylenediaminetetraacetic acid (EDTA), the X-ray diffraction intensity decreases markedly, and the amorphous fraction increases.^80^ Consistently, ^31^P solid-state NMR revealed that the chemical environment of pSer residues shifts from a rigid crystalline state to a high-mobility hydrated state after EDTA treatment. Remarkably, adding Ca^2+^ restores the original rigid state. These results clearly show that the repetitive pSer sequences form reversible β-sheet crystals *via* metal-phosphate bridging and β-turn structure formation in caddisfly silk (Fig. 6C). This dynamic crystalline network directly underpins the mechanical properties of caddisfly silk. In tensile tests, native caddisfly fibers are extensible and display a distinct yield point followed by a stress plateau, as the Ca^2+^-pSer cross-links progressively fragment under load.^83^ Upon unloading, the broken cross-links reform through Ca^2+^ coordination, which allows the β-sheet nanocrystals to assemble and the fibers to fully recover their original stiffness and length.^84^ This reversible sacrificial bonding mechanism dissipates mechanical energy and enables repeated shock mitigation. As a result, caddisfly silk is exceptionally tough, and self-healing experiments have shown that it can recover approximately 99% of its initial tensile strength and stiffness within hours after deformation.^84^ In other words, caddisflies make sophisticated use of phosphorylation as a PTM to construct a fiber-reinforced molecular architecture underwater. This strategy is thought to have been acquired when the caddisfly lineage adapted from terrestrial to aquatic habitats, and crystallization mediated by pSer represents an original evolutionary solution.

#### 3.3.2. PTMs in the underwater adhesive coating

The silk extruded by caddisfly larvae consists of the fibrous core described above and an adhesive outer coating that covers the fiber surface.^77^ Functionally, this outer layer corresponds to silkworm sericin.^85^ It unites the fibers and mediates adhesion to substrates such as stones and leaves. Molecularly, however, this coating is entirely different from sericin and is composed of proteins and enzymes specialized for the aquatic environment. First, proteins in the outer layer are enriched in acidic residues such as Glu and Asp, forming highly hydrophilic glycoproteins with an overall negative charge. Although the detailed glycan structures remain unresolved, lectin staining with wheat germ agglutinin (WGA) indicates the presence of glycans such as GalNAc. Notably, this adhesive layer contains a peroxidase. In the silk glands of caddisfly larvae, a heme peroxidase (peroxinectin (Pxt))^86^ is expressed as a component of the adhesive layer, and MS confirmed that this enzyme is a glycoprotein bearing *O*-linked glycans. Pxt is secreted during silk spinning and catalyzes the oxidative crosslinking of Tyr residues. Through the activity of Pxt, diTyr cross-links form within the adhesive layer, converting the outer layer into an insoluble network. Interestingly, Pxt on the outer layer also crosslinks with environmental polyphenols. In fact, when fluorescein-labeled synthetic polyphenols are coated on an underwater substrate, Pxt covalently links those polyphenols to the outer layer of the silk. Thus, the caddisfly silk adhesion strategy is two-pronged: physical crosslinking *via* phosphorylation and crosslinking *via* Pxt.^87^ The former contributes to the toughness and elasticity of the fiber core, while the latter ensures the strength and water resistance of the adhesive layer. Moreover, the latter strategy resembles the DOPA-based adhesion observed in organisms such as gall midges, barnacles, and cone snails, representing a convergent solution for underwater adhesion. Indeed, the marine polychaetes *Phragmatopoma* spp. use phosphorylated proteins for underwater adhesion, suggesting that the combination of phosphorylation and catechol chemistry is a widely adopted strategy among aquatic organisms.^88^ Research on PTMs in caddisfly silk is relatively recent and has attracted attention as a treasure trove for biomimetic materials.^89,90^

### 3.4. PTMs of other insect silks

Beyond spiders and silkworms, many other insects produce silk-like protein fibers.^91^ Bagworm larvae build tubular cases by stitching together small twigs and leaf fragments with their own silk. Bagworm silk presumably consists of large proteins, but the details have not been well studied. Recent transcriptomic analysis of *Eumeta variegata* revealed an extremely long, repetitive silk fibroin gene (19 kbp) encoding a Gly/Ala-rich protein, supporting the presence of heavy-chain fibroin analogs in bagworms.^92^ Given that it is used to build shelters and is exposed to wind and rain in terrestrial environments, it is possible that bagworm silk undergoes phenolic crosslinking. Indeed, the interior of bagworm cases sometimes shows brown discoloration and hardening of the threads, which may result from reactions between plant-derived tannins or microbially degraded lignin components.^93^

Many hymenopteran larvae, including numerous species of bees, wasps, and ants, spin cocoons during their final instar stage before pupation.^94,95^ These Hymenopteran silks have distinct origins from Lepidopteran fibroin and can possess properties akin to those of cuticular proteins.^96^ Particularly noteworthy is the cocoon thread of the willow sawfly, *Nematus oligospilus*, which was recently reported to be an entirely new type of silk that forms a collagen-like triple helix.^97^ Sutherland and colleagues reported that the *N. oligospilus* cocoon comprises three small collagen proteins that carry Gly-Pro-Pro repeats and self-assemble into triple-helical fibers. Strikingly, despite being an insect, Hyp was detected at Pro residues in this type of collagen silk. The resulting willow-sawfly cocoon is a novel material that combines tensile strength with flexibility. However, few detailed analyses have been performed on other Hymenopteran silks, but these proteins tend to be relatively large and rich in Gly and Tyr.^98^ For instance, the cocoon silks of nonsocial wasps (*Ichneumonoidea*) are fibroin-like and composed largely of Gly, Ala, and Ser residues.^98^

Overall, PTMs of insect silks are extraordinarily diverse and have been optimized for each ecological niche. Unique PTM strategies not found in silkworms and spiders have also evolved; for instance, the adoption of a collagen in Hymenoptera and hybrid crosslinking with plant polyphenols in bagworms and caddisflies. A comprehensive survey of PTMs in less-studied silks should allow a holistic understanding of the gamut of material strategies devised by life and their translation into human use.

## 4. Applications to materials science

### 4.1. Introducing PTMs *via* synthetic biology and chemical synthesis approaches

**Fig. 7:**
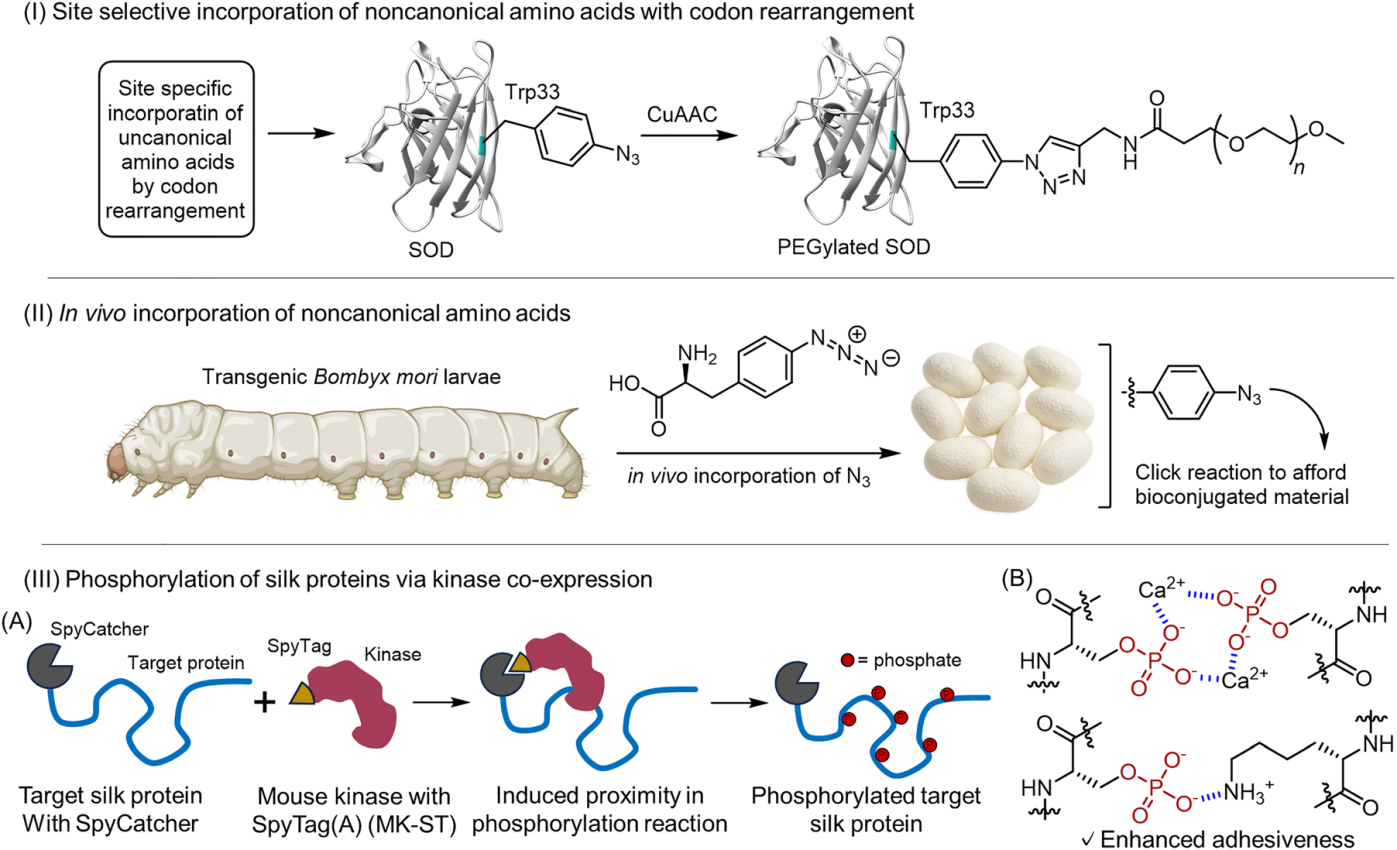
Strategies for engineering PTMs into structural proteins. (I) Site-selective incorporation using codon rearrangement: genetic codon editing introduces azido-phenylalanine at Trp residues, followed by click conjugation with PEG.^105^ (II) *In vivo* incorporation of noncanonical amino acids: transgenic silkworms fed azido-phenylalanine produce silk fibroin carrying azido groups.^107^ (III) (A) Induced proximity approach for silk protein phosphorylation. Schematic representation of aggregate spider silk. (B) Scheme of a possible adhesion mechanism of phosphorylated silk supplemented with Ca^2+^ ions.^108^ Adapted from ref. 108 under the Creative Commons CC BY 4.0 license.

By strategically manipulating the PTMs of structural proteins, we can finely tune their material properties, including mechanical strength, extensibility, thermal stability and heat resistance, water resistance, and self-healing.^99^ To append PTMs to material proteins, a combination of synthetic-biological and chemical approaches is indispensable.^100^ Advances in genetic engineering have allowed the introduction of artificial modification acceptor sites into protein primary sequences. A classic example is the incorporation of unnatural amino acids (UAAs) *via* genetic code expansion and amber codon reassignment.^101,102^ Using such techniques, we can insert UAAs bearing novel functional groups at defined positions in a protein. Examples include alkenes, azides, alkynes, ketones, and phenylalanine derivatives. This approach endows the material with chemical reactivities that are absent in biological systems. Notably, natural protein materials often contain nonstandard amino acids that are PTM products, which are crucial for function. However, conventional expression hosts such as *E. coli* often cannot install these PTMs. Introducing artificial PTMs by genetically encoding UAAs has thus become a powerful approach. Recent work has achieved genetic code expansion to install DOPA into protein polymers.^103^ Catechols are the adhesive residues in mussel adhesive proteins. By introducing catechols into engineered proteins, we can design high-performance materials with underwater adhesion and self-healing capabilities.^104^ In this way, genetic methods for incorporating UAAs and PTM-like modifications are dramatically expanding the repertoire of protein–material design. Bioorthogonal chemistry also provides powerful artificial modification routes. Cell-permeable reagents can be conjugated to UAAs *via* click reactions, enabling covalent attachment of polymers. For instance, *p*-azidophenylalanine introduced by genetic encoding can undergo a click reaction with alkyne-bearing compounds to append polyethylene glycol (PEG) chains, improving the durability and water retention of protein hydrogels (Fig. 7I).^105^ Other reports introduce UAAs bearing spiro moieties or thiol groups and induce gelation by the specific crosslinking with externally added crosslinkers.^106^ While the applications of these techniques to the modification of silk proteins remain limited, they are expected to become important tools.

Beyond amber codon reassignment, residue-specific UAA incorporation has also been realized in multicellular systems. For example, Kojima and Teramoto demonstrated that transgenic silkworms fed phenylalanine analogs could produce silk fibroin carrying azide groups, yielding clickable silks that survive degumming and enable versatile bioorthogonal modifications (Fig. 7-II).^107^

Another recent approach uses proximity-assisted enzymatic PTM installation. Using a SpyCatcher/SpyTag system, silk proteins expressed in *E. coli* can be selectively recruited to a coexpressed kinase, greatly enhancing on-target phosphorylation while minimizing global toxicity (Fig. 7III(A)).^108^ This simple design enables high-density, multisite phosphorylation of dragline and aggregate silk, yielding recombinant phosphorylated silks with measurably improved adhesion and Ca^2+^-dependent cohesion properties (Fig. 7III(B)). Because the scaffold is modular, the same strategy could be adapted for other PTM enzymes to program PTM patterns in engineered fibrous proteins.

**Fig. 8:**
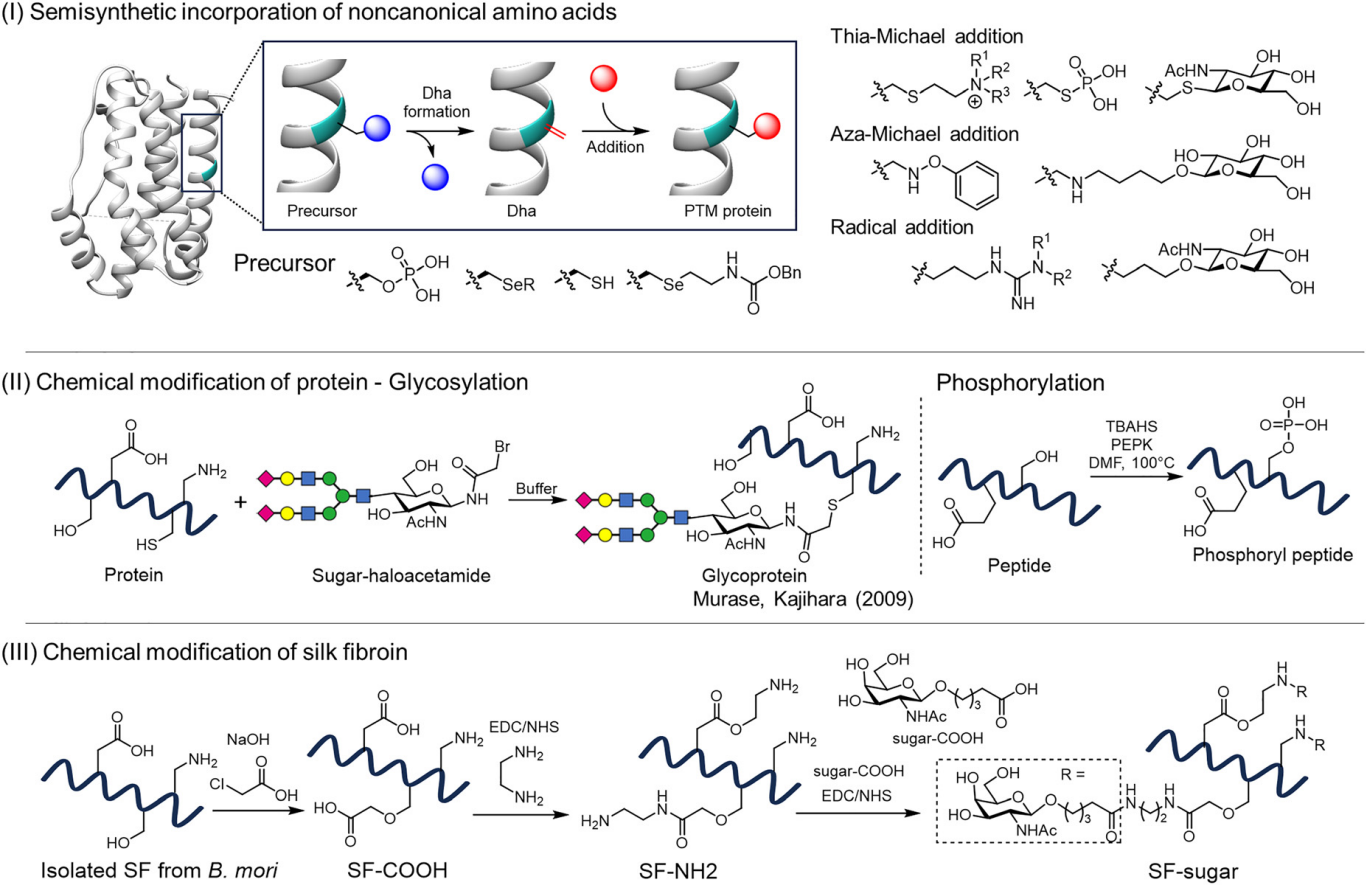
Chemical strategies for introducing PTMs into proteins. (I) Semisynthetic incorporation of noncanonical amino acids: precursor residues are genetically introduced and subsequently converted to dehydroalanine (Dha), which serves as a reactive handle for Michael addition or radical-based modification to install diverse PTMs.^107^ (II) Chemical modification of proteins and peptides: (a) site-selective glycosylation *via* the coupling of haloacetamide-activated glycans to Cys residues;^112,113^ and (b) chemical phosphorylation targeting Ser/Thr residues to overcome the challenge of competing nucleophiles such as Lys.^114,115^(III) Chemical modification of silk fibroin: global conjugation of sugars to carboxyl, amino, or hydroxyl groups on fibroin, yielding glycosylated silk materials.^116^

Furthermore, dehydroalanine (Dha) chemistry has been shown to be a versatile tag-and-modify platform for installing a wide spectrum of PTMs. These include phosphorylation, glycosylation, ubiquitination, and acetylation, all of which can be introduced into recombinant proteins under mild conditions (Fig. 8I).^109^ Such chemical methods are extremely powerful because they endow materials with functional groups that biology does not natively create, bridging synthetic polymer chemistry with protein material approaches.

Rapidly advancing cell-free systems and secretion-based expression platforms are also useful for PTM installation. Cell-free protein synthesis (CFPS)^110^ using cell extracts or purified transcription-translation machinery eliminates the constraints of cell walls and viability, enabling synthesis with high concentrations of UAAs.^111^ CFPS also allows the coaddition of enzymes to install PTMs concurrently with translation. Indeed, studies using CFPS to macrocyclize RiPPs (ribosomally synthesized and post-translationally modified peptides) or to append artificial glycans to proteins have screened many enzyme variants within hours and identified mutants with superior glycosylation efficiency.^112^ By combining CFPS with high-throughput analysis, the process of PTM installation can now be rapidly optimized.^113^ Conversely, secretion using eukaryotic cell culture can favor spontaneous disulfide formation and near-native glycosylation by routing products through the secretory pathway. Efforts are underway to secrete spider silk proteins from yeast to obtain materials in which the stabilizing crosslinks required for higher-order structures are correctly formed.^37^

In addition, natural protein materials can be chemically modified after expression or synthesis. Elegant examples include the work of Kajihara and colleagues, who developed haloacetamide-based Cys-selective coupling to construct homogeneous glycoproteins with defined saccharide structures (Fig. 8II, left panel).^114,115^ Moreover, Kanai and colleagues recently reported catalytic phosphorylation of unprotected Ser/Thr and Tyr residues (Fig. 8II, right panel).^116,117^ Similarly, mucin-inspired engineering of silk fibroin revealed that covalent conjugation of sugars such as GalNAc to the side chain functionalities of Lys, Ser, Thr, Asp, and Glu inhibited *Streptococcus mutans* biofilm formation while preserving bacterial growth, effectively reproducing the antivirulence effect of mucin (Fig. 8III).^118^ This approach imparts new functions without re-expression, although the lack of site specificity and chemoselectivity yield heterogeneous modifications. Collectively, combining synthetic biological tools with chemical methods makes freely programmable PTM of silk protein design a practical reality. This approach opens paths to materials whose functions are absent in nature and to improved biomaterials.

Despite these advances, several limitations remain in current strategies for installing PTMs in silk proteins. Genetic code expansion and synthetic biology approaches can be constrained by incorporation efficiency and scalability, and may impose metabolic burden or toxicity in host organisms, particularly when introducing multiple or high-density modifications. Chemical modification strategies introduce heterogeneity in modification patterns and are difficult to control at precise stoichiometry. Addressing these challenges will be essential for translating programmable PTM strategies toward scalable and structurally well-defined silk-based materials.

### 4.2. Discovering unknown protein PTMs

The discovery and comprehensive understanding of novel PTMs directly propel the field of structural protein materials. Recent advances in proteomics have established highly sensitive MS methods for identifying low-abundance and previously unknown PTMs that were once difficult to detect.^119^ In particular, data-independent acquisition (DIA) enables comprehensive capture of peptide fragments in a sample with precise quantification and is being vigorously applied to PTM analysis.^120^ Because DIA acquires spectra without bias, modified peptides are recorded exhaustively, allowing site-specific global analyses of the phosphoproteome or glycoproteome.^121,122^ By combining DIA with deep learning-based *in silico* spectral libraries and scoring, the accuracy of PTM identification has improved dramatically.^123^ This now permits high-throughput, high-accuracy workflows that span detection, site localization, and characterization of combined modifications.

Furthermore, accelerated and parallelized cross-linking mass spectrometry (XL-MS) is transforming the study of structural proteins.^124^ XL-MS covalently crosslinks proximal residues within proteins using reactive reagents for MS-based identification of the resulting cross-linked peptides. This approach reveals protein structures and interaction networks in solution. Recent work has succeeded in crosslinking entire cells to obtain proteome-wide distance constraints under native intracellular conditions. Bartolec reported the largest dataset to date, identifying 28 910 unique residue pairs derived from 91 709 cross-linked spectral matches across 4084 human proteins and 2110 protein–protein interactions.^125^ Importantly, these crosslinks capture proteins with their natural sequences, PTM states, subcellular locations, and cofactor-bound forms. They provide physiological snapshots that cannot be obtained with purified proteins alone. XL-MS data also serve to validate and refine machine learning structure predictions such as AlphaFold2,^126^ ushering in an era of systematic structural proteomics. Together, DIA-based proteomics and XL-MS provide a powerful platform for discovering unknown PTMs and clarifying their structural roles.

To identify unknown PTMs, it is also crucial to learn from diverse organisms and ecologies. Modifications rare in a single species may emerge through cross-species comparisons. Researchers worldwide are accumulating PTM data from many organisms, and PTM databases are being continuously updated to organize this vast amount of information. A recent example is dbPTM 2025, a comprehensive resource that integrates >2.79 million experimentally validated PTM sites from 48 data sources and over 80 000 publications.^127^ dbPTM 2025 supports searches by species, PTM type, and modified residue. With an eye to disease research, the database also integrates large-scale cancer phosphoproteomics and kinase activity profiles, supporting PTM network analyses and comparisons across species and diseases. The development of such cross-species, comprehensive PTM databases is expected to greatly accelerate the identification of unusual PTMs and the understanding of their evolutionary conservation and diversity.

Applications of artificial intelligence (AI) to PTM research are likewise advancing rapidly. While deep learning structure prediction revolutionized the inference of protein structures, it initially ignored PTMs.^128^ Current efforts are underway to overlay PTM data onto predicted structures to evaluate changes in stability and to visualize structural fluctuations in PTM-bearing regions. In protein sequence design with generative AI, attention is turning to PTMs. The recently reported PTM-aware protein language model “PTM-Mamba,” trained on approximately 79 000 modified protein sequences (based on >3 10 000 PTM records), successfully embeds PTM-bearing sequences into high-dimensional spaces that capture modification-specific features.^129^ Unlike earlier language models that masked or ignored modified residues, PTM-Mamba encodes modified amino acids as dedicated tokens and integrates embeddings from large models such as ESM-2, enabling the model to represent functional and structural changes associated with PTMs. Visualization of the embedding space shows modified and unmodified versions of a protein located nearby yet separable, with clustering by PTM type. This is the first demonstration that deep learning can learn and represent the effects of PTMs on structure and function, opening the door to applications such as designing stabilizing mutations contingent upon a target PTM or predicting crosstalk among multiple PTMs. Complementary efforts include prompting Transformer-type models to predict PTM sites (PTMGPT) and, conversely, generating sequences expected to realize specified PTM patterns.^130^ AI excels at discovering hidden regularities in massive datasets. Data-driven PTM design support is now at the forefront, enabling discovery of unknown PTMs and prediction of surprising PTM combinations and their functions. However, applications of these AI-based approaches to silk PTMs remain limited at present, and most current efforts are still at a conceptual or exploratory stage. Future AI-driven approaches will become particularly valuable in addressing unresolved questions in silk PTMs. For example, quantitative prediction of phosphorylation site occupancy and stoichiometry from sequence and proteomic data could help distinguish structurally dominant modifications from low-abundance modifications. In addition, integrative computational frameworks combining proteomics, structural modeling, and materials simulations could clarify how specific PTMs influence solubility and assembly during spinning.

## Outlook

Protein–materials science centered on PTMs holds immense promise at the interface of life science and materials science. PTMs are molecular switches through which cells rapidly alter protein states in response to their environment. Leveraging them in material design enables new functional materials to respond to external cues. Although PTM research is rapidly expanding across molecular and cellular biology, our understanding of PTMs in silk proteins remains incomplete. Their diversity, context dependence, and often low stoichiometry make systematic characterization difficult, leaving many fundamental questions unresolved. Precisely controlling and observing PTM reactions within artificial materials could deepen our understanding of *in vivo* PTM networks. Through collaboration between data-driven computation and experimentation, the creation of intelligent protein materials with PTMs woven in at will could lead to transformative breakthroughs. Consequently, the fusion of materials and life sciences will serve as the driving force behind future advances in this field, establishing a new interdisciplinary frontier.

## Author contributions

K. N. and K. N. wrote and reviewed the manuscript.

## Conflicts of interest

There are no conflicts of interest to declare.

## Data Availability

No primary research results, software or code have been included and no new data were generated or analysed as part of this review.
